# Reflectance-Based Organic Pulse Meter Sensor for Wireless Monitoring of Photoplethysmogram Signal

**DOI:** 10.3390/bios9030087

**Published:** 2019-07-10

**Authors:** Fahed Elsamnah, Anubha Bilgaiyan, Muhamad Affiq, Chang-Hoon Shim, Hiroshi Ishidai, Reiji Hattori

**Affiliations:** 1 Department of Applied Science for Electronics and Materials, Kyushu University, Fukuoka 816-8580, Japan; 2 COI STREAM, Center for Organic Photonics and Electronics Research (OPERA), Kyushu University, Fukuoka 819-0395, Japan; 3 Konica Minolta, Inc., Ishikawa-cho, Hachioji 192-8505, Japan; 4 Global Innovation Center (GIC), Kyushu University, Fukuoka 816-8580, Japan

**Keywords:** organic optoelectronic device, pulse meter, biosensor, Bluetooth low energy (BLE), photoplethysmogram (PPG)

## Abstract

This paper compares the structural design of two organic biosensors that minimize power consumption in wireless photoplethysmogram (PPG) waveform monitoring. Both devices were fabricated on the same substrate with a red organic light-emitting diode (OLED) and an organic photodiode (OPD). Both were designed with a circular OLED at the center of the device surrounded by OPD. One device had an OLED area of 0.06 cm^2^, while the other device had half the area. The gap distance between the OLED and OPD was 1.65 mm for the first device and 2 mm for the second. Both devices had an OPD area of 0.16 cm^2^. We compared the power consumption and signal-to-noise ratio (SNR) of both devices and evaluated the PPG signal, which was successfully collected from a fingertip. The reflectance-based organic pulse meter operated successfully and at a low power consumption of 8 µW at 18 dB SNR. The device sent the PPG waveforms, via Bluetooth low energy (BLE), to a PC host at a maximum rate of 256 kbps data throughput. In the end, the proposed reflectance-based organic pulse meter reduced power consumption and improved long-term PPG wireless monitoring.

## 1. Introduction

**Figure 1:**
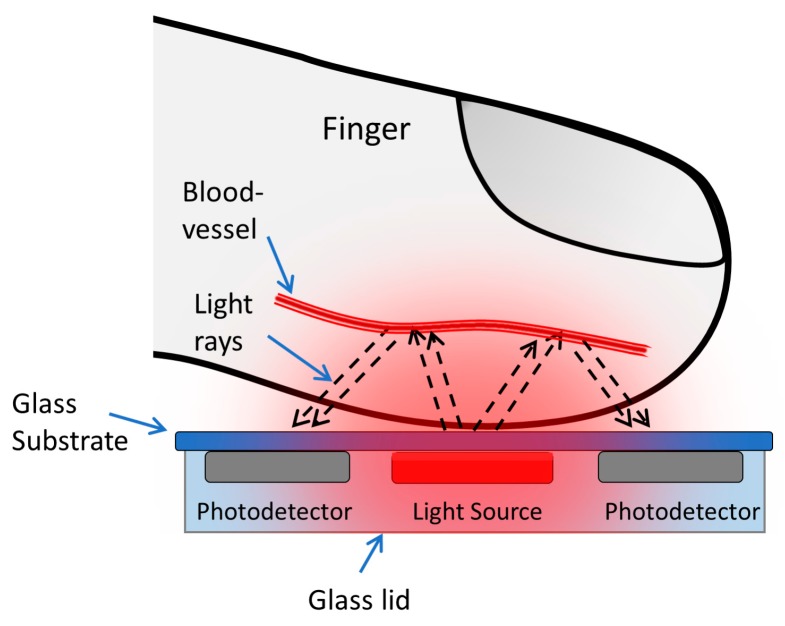
Acquisition of photoplethysmogram (PPG) signal from the reflection method.

**Figure 2:**
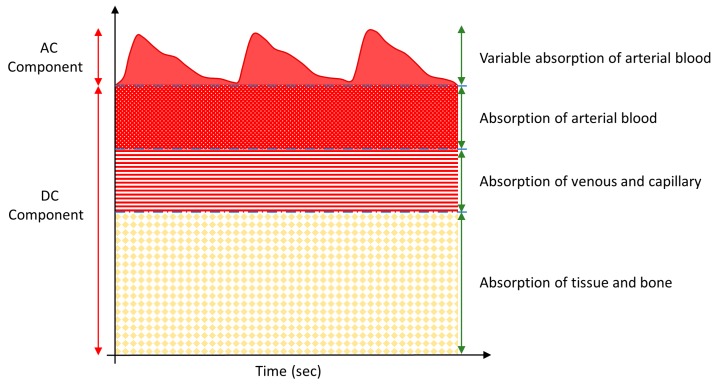
Schematic of light absorption in body tissues.

A pulse meter is a device used to measure the rate of rhythmic contraction and expansion of an artery at each beat of the heart based on the photoplethysmogram (PPG) principle. It has received enormous attention over the past decade, primarily from the healthcare industry, due to its continuous, real-time, and noninvasive monitoring, which provides the information necessary to determine an individual’s health status and even provide a preliminary medical diagnosis [1,2,3]. Pulse meters rely on the PPG principle, which necessitates a light source and a light detector. The light is transmitted through tissue and reflects onto the light detector, as shown in Figure 1. When the heart beats, the blood volume of the arteries changes accordingly and causes variable light absorption, allowing changes in reflected light to be detected as a PPG signal. The detected PPG signal comprises an alternating (AC) component, due to the variable absorption of the pulsatile arterial blood, and a steady-state (DC) component, from the veins, capillaries, tissues, bones, and other non-pulsatile components, as shown in Figure 2 [4]. The AC component is the outcome of light absorption by the arteries, while the DC component is the outcome of light absorption by body tissues and veins. Therefore, the pulsatile effect occurs only in the arteries, not in the veins or other non-pulsatile components. There are two approaches that can be used to obtain a PPG signal from a biosensor pulse meter: reflection and transmission. The reflection method was utilized in this work because of the freedom of use. The device could be easily worn or attached to different parts of the human body. The transmission method involves tissue transillumination and required that a light source and a detector be placed opposite each other. Consequently, the transmission method could only be used on external body parts such as fingertips and ear lobes.

In recent years, organic pulse oximeters have received significant attention from researchers due to the many advantages of organic optoelectronic devices, including their relative low cost, simple fabrication and their ability to be fabricated on flexible substrates, for comfortable wearable medical devices. Furthermore, large organic photodiodes (OPDs) can be easily fabricated, compared to the restricted size of generic silicon-based photodiodes (PD). This has made organic light-emitting diodes (OLEDs) and OPDs preferable for use in wearable pulse oximeters [5,6,7]. In the literature, there were several proposed OLED and OPD designs that aimed to improve power consumption and signal quality. To improve the longevity of the batteries in inorganic reflective pulse oximeters, the authors of [8] proposed an annular PD ring design with a light-emitting diode (LED) located at the center. A rectangular OPD device and a device with two separated square OLEDs were proposed in [9]. Meanwhile, the authors in [10] proposed a design with a circular OPD in the center of a half-ring of red polymer light-emitting diodes (PLEDs) and a half-ring of green PLEDs. The authors of [11] conducted optical simulations to test the power consumption of their designs involving a ring of OPDs surrounding a circular OLED. Various other researchers have attempted to develop a wireless pulse meter and to solve the problems associated with it, such as signal quality and power consumption. In [12], the study proposed a compact portable module composed of an array of photodetectors that could be distributed radially around LEDs and the PPG signal sent via a Zigbee protocol wireless module. The chip consumed 38 mA to transmit the data and 37 mA to receive the data. The red LEDs consumed about 38 mW and the IR LEDs about 26 mW. The authors of [13] proposed a wireless heart rate (HR) and peripheral oxygen saturation (SpO_2_) monitoring system that could be connected to a local wireless network via Wi-Fi technology and the information was transmitted in real time to a webpage for remote monitoring. The current consumption of that wireless microcontroller unit (MCU) chip was 229 mA for transmission (TX) traffic and 59 mA for reception (RX) traffic. Other researchers proposed wireless pulse oximeters but did not mention the power consumption of the proposed device, such as [14], who proposed a wireless ring-type pulse oximeter with multiple detectors for sending the signal to the host system via Bluetooth. In [15], the authors presented a PPG wireless monitoring device embedded in a hat and glove that could send the signal via Bluetooth. However, the wireless PPG signal quality was not adequately addressed in the previous research and prototypes. Moreover, power consumption is a top priority in the development of wireless pulse meters because they are battery operated. Although the previous works on pulse oximeters required two light sources, which consumed double the power of one light source. The previously proposed wireless devices remained impractical for long-term use, although the pulse oximeters required two light sources, which consumed double the power of one light source. Therefore, miniature, portable, wearable pulse meters that are able to be monitored wirelessly will provide more freedom and comfort and will be more compatible with conventional devices, which will lead to simplified health monitoring [16]. In terms of organic optoelectronic devices, the material structure, dimension design, and the characteristics of the OPDs and OLEDs in this paper were different to those in previous works. Here, we propose different material structures and dimensions of OLED/OPD devices based on our previous works [17,18,19] as part of our continued attempts to improve the power consumption and signal quality of organic pulse oximeter. 

In this work, we compared two different organic pulse meter designs using OLEDs and OPDs. We evaluated their performances in terms of the power consumption of the wireless pulse meter. This paper highlighted the importance of designing OPD and OLED structures, guided by optical simulation, to enhanced signal quality and minimize the power consumption for monitoring the PPG waveforms via Bluetooth low energy (BLE).

## 2. Materials and Methods

Two devices, Device-1 and Device-2, were fabricated with different OLED areas. Both devices had a circular OLED at the center and were surrounded by a ring of OPDs. Device-1 had an OLED area of 0.06 cm^2^ while Device-2 had an OLED area of 0.03 cm^2^. The gap distance between the OLED and OPD was 1.65 mm and 2 mm for Device-1 and Device-2, respectively. Both devices had the same OPD area of 0.16 cm^2^. The OPDs were ring-shaped and surrounded the OLED to effectively collect the reflected photons from the skin.

### 2.1. Optical Simulation

**Figure 3:**
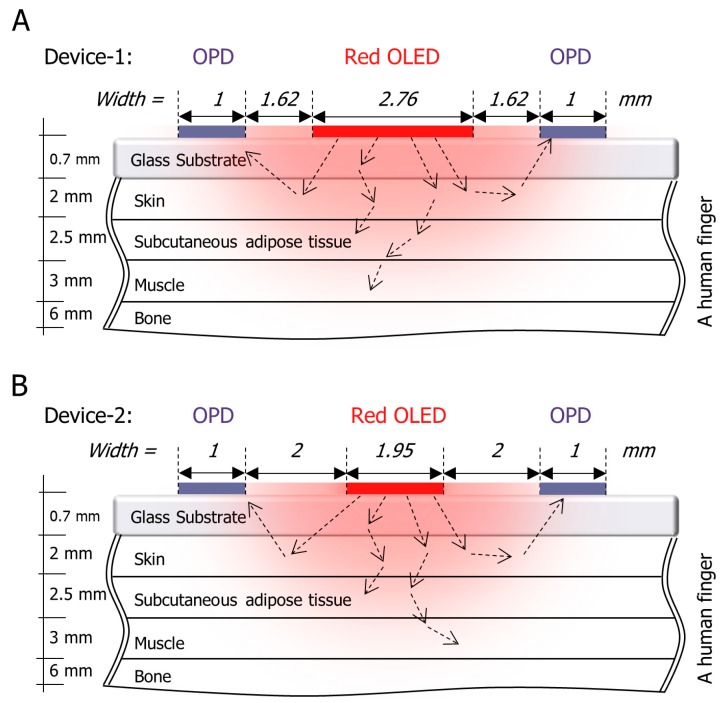
A simplified finger schematic with the organic light-emitting diode (OLED) as the light source and the organic photodiodes (OPDs) as the surface receiver for (**A**) Device-1 and (**B**) Device-2.

To simulate the whole system optically, a simplified finger model was designed and configured, followed by the OLED and OPD device models. Next, all models were simulated optically using LightTools software (Synopsys, Inc., Mountain View, CA, USA), which uses the ray-tracing and Monte Carlo methods. Figure 3 illustrates the distribution of the light rays from the red OLED into the human finger and shows the simplified four-layer structure of a finger model. The red OLED was adopted due to its advantages over other OLEDs colors including the green OLED and near-infrared (NIR) OLED. In the case of green OLED, the green light has a shorter wavelength, which leads to decreasing the penetration depth of the light in the human body and getting absorbed. In the case of the NIR OLED, the NIR region of the spectrum has a longer wavelength, which leads to increasing the penetration depth of the light in the human body. However, the EQE in this region is significantly lower than the OLED in the visible region. Moreover, fabricating NIR OLED/OPD is difficult and still under research and development [20,21]. In the model, the arterial blood vessels were assumed to be included in the skin and the subcutaneous adipose tissue for simplicity. Device-1 and Device-2 were attached to the simplified finger model as shown in Figure 3A,B. The optical parameters were approximated using the literature [22,23,24,25], and are shown in Table 1 where the refractive index of the material describes how fast light propagates through the material. The Henyey–Greenstein function was used to approximate the angular scattering dependence of single scattering events in biological tissues.

**Figure 4:**
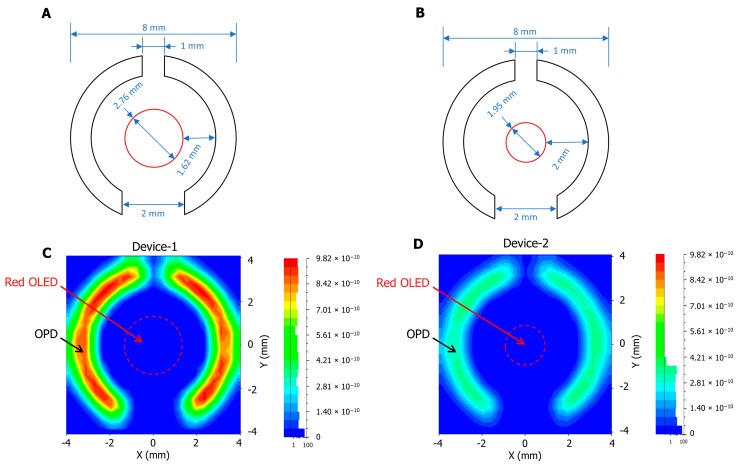
The dimensions of the OLED and OPD design structure in (**A**) Device-1 and (**B**) Device-2. The optical simulation results at the receiver’s surface in Watt per mm^2^ for (**C**) Device-1 and (**D**) Device-2.

The dimensions of the OLED and OPD design structure for both devices are illustrated in Figure 4A,B, which demonstrates that the area of the OLED in Device-1 was designed to be double the size of that in Device-2 in order to evaluate the effect of increasing the light source and decreasing the gap distance between the OLED and the OPDs. The radiant power of the light source was assumed, based on our previous red OLED devices (625 nm), to be 2.8 μW and 1.4 μW for Device-1 and Device-2, respectively. The simulation traced one million rays in each device. The simulation results, shown in Figure 4C,D, show the irradiance on the OPD area, where the maximum estimated irradiance for Device-1 was 9.8 × 10^−10^ W/mm^2^ and for Device-2 was 3.7 × 10^−10^ W/mm^2^. While the simulation presented an unequal irradiance distribution due to the spline effect of the ray simulation, we assumed the maximum irradiance was distributed equally. Therefore, the total received power of Device-1 and Device-2 was 15.6 × 10^−9^ W and 5.9 × 10^−9^ W, respectively. To put that into perspective, 0.55% of light rays reflected on the OPD of Device-1, while Device-2 received 0.42% of the light rays on the OPD. In these simulation results, when the gap between the OLED and the OPDs was decreased from 2 mm in Device-2 to 1.62 mm in Device-1, the total power increased to more than double, which means that the amplitude of the PPG in Device-1 was expected to be bigger than the amplitude in Device-2.

### 2.2. The Organic Optoelectronic Device

**Figure 5:**
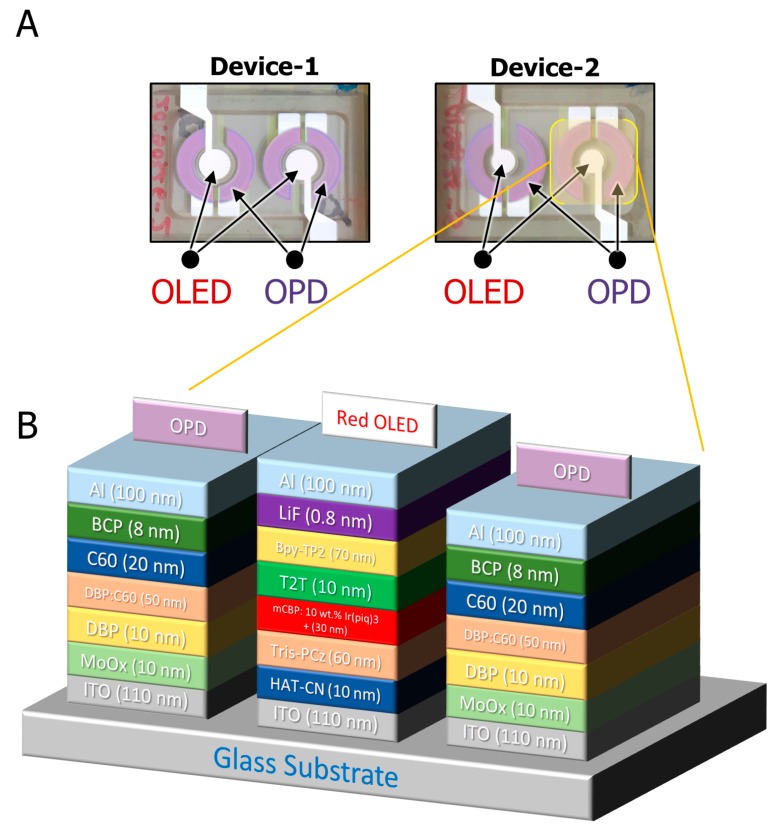
(**A**) The fabricated organic biosensor pulse meter for Device-1 and Device-2. (**B**) A cross-section of the organic material structure of the OLED and OPD for both devices.

Device-1 and Device-2 were fabricated with two different OLED sizes and a similar OPD area. As a monolithic device, the OLED and the OPD were deposited onto the same 0.7-mm-thick glass substrate. For simplicity of the structure of the device and future fabrication, identical red OLED and OPD structures, shown in Figure 5A, were fabricated on the same substrate. The organic material structure of the OLEDs and OPDs were the same for Device-1 and Device-2, as shown in Figure 5B.

The fabrication process of the organic pulse meter sensor started with the preparation of glass substrates that were coated with indium-tin-oxide (ITO) and then loaded into an evaporator chamber to deposit organic metal layers with the proper shadow mask patterns for the OLED and followed by the OPD. The shadow masks were designed in a way to prevent the issue of misalignment in the multi-mask processing of monolithic device fabrication. Then, the samples were encapsulated without exposure to air in a glove box, which was connected to the deposition chamber by glass lid using UV curable epoxy resin [18]. Thereafter, the samples were used for evaluating the performance of the pulse meter sensor. Figure 5B illustrates the structure of the OLED and OPD used in Device-1 and Device-2. The OLED device structure consisted of ITO (110 nm), a transparent anode that allowed the generated light to be emitted from the glass panel, HAT-CN (10 nm) as the hole injection layer, Tris-PCz (60 nm) as the hole transport layer, mCBP:10 wt% Ir(piq)_3_ + (30 nm) as the emissive layer where the holes and electrons recombined to emit light, T2T (10 nm) as the electron blocking layer, Bpy-TP2 (70 nm) as the electron transport layer, LiF (0.8 nm) as the electron injection layer, and Al (100 nm) as the cathode for the OLED. The OPD structure consisted of ITO (110 nm), with MoO_x_ (10 nm) as the hole injection layer, DBP (10 nm), DBP:C60 (50 nm), and C60 (20 nm) combined in the device active layer, BCP (8 nm) as the electron transport layer, and Al (100 nm) as the cathode.

**Figure 6:**
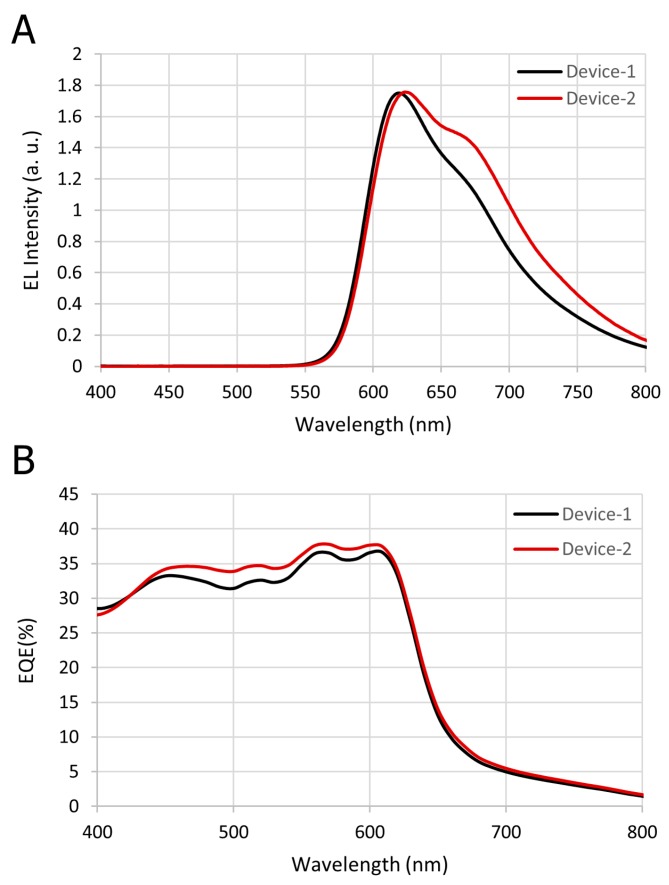
(**A**) The OLED’s electroluminescence spectrum with respect to wavelength (nm). (**B**) The OPD’s external quantum efficiency (EQE) with respect to the wavelength.

The characteristics of the OLED and OPD devices were essential to the signal quality of the biosensor pulse meter. Organic LEDs have many advantages over LEDs, despite the fact that they have a shorter lifetime. OLEDs are relatively easy to produce, flexible, foldable, transparent, and capable of fast switching. Moreover, their cost is lower and they consume less power. The emission spectra of the OLED for Device-1 and Device-2, where the maximum intensity of light was 625 nm, are shown in Figure 6A. The slight difference in the characteristics of both devices was due to thickness variation because the devices were not fabricated at the same time. Figure 6B presents the OPD’s external quantum efficiency (EQE) at zero-bias condition, where the maximum EQE was about 37% at 625 nm, which sufficiently overlapped with the peak EL of the red OLED wavelength.

### 2.3. The Device Structure

**Figure 7:**
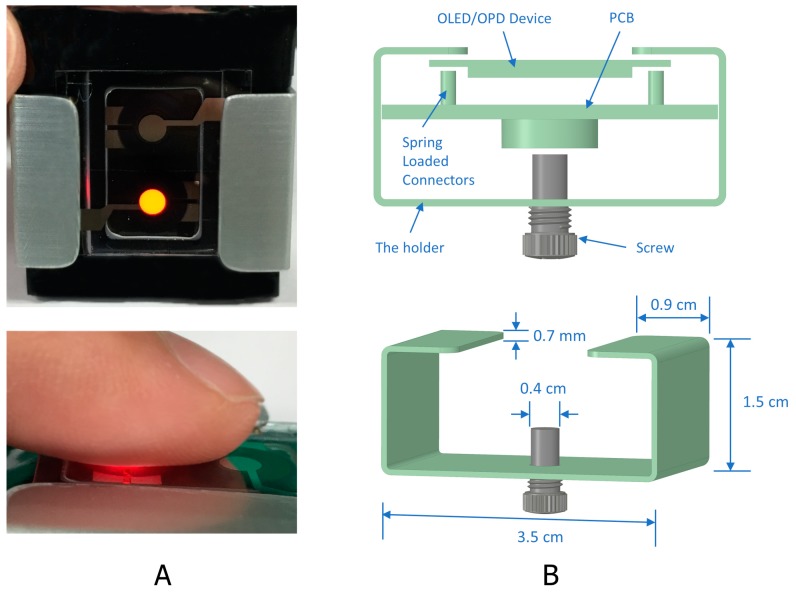
(**A**) The proposed portable pulse meter prototype for wireless monitoring via Bluetooth low energy (BLE). (**B**) The device holder, that used to fix the parts together, and its dimensions.

The structure of the proposed biosensor pulse meter for wireless monitoring was composed of three parts: an organic optoelectronic device, a device holder, and a PCB that included a driver circuit and a wireless microcontroller unit (MCU) module, as illustrated in Figure 7.

In the driver circuit, the small photocurrent generated by the OPD needed a transimpedance amplifier (TIA) to convert the OPD current to a voltage signal. Next, the PPG signal required a filtration process to eliminate the high-frequency noise and the DC component from the signal. The last stage was to amplify the AC component of the PPG signal and send it through an analog-to-digital converter (ADC) process. Then, the PPG signal was transmitted via serial communication block (SCB) or Bluetooth low energy (BLE). The analog circuit employed in this work was previously described, see [17] for more details. Figure 7A presents the portable pulse meter prototype that was proposed for wireless monitoring of the PPG signal, and Figure 7B illustrates the dimensions of our novel tool for fixing the biosensor pulse meter and the PCB, including the driver circuit and wireless MCU together with aluminum shield for further protection. The wireless MCU module (CYBLE-214015-01, Cypress Semiconductor, San Jose, CA, USA) implemented in this work is a certified and qualified module supporting BLE wireless communication. It uses a BLE technology in which the battery life is enhanced by keeping the radio activity short and allowing the device to reside in standby or power-down mode during most of its operating time to consume less power.

## 3. Results and Discussion

### 3.1. Comparative Results for Device-1 and Device-2

**Figure 8:**
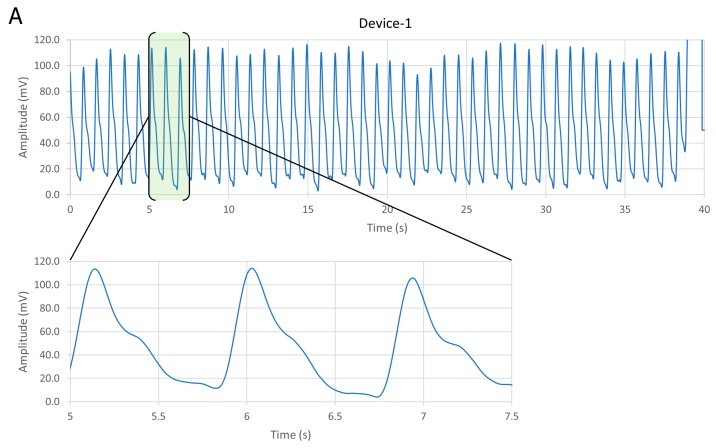

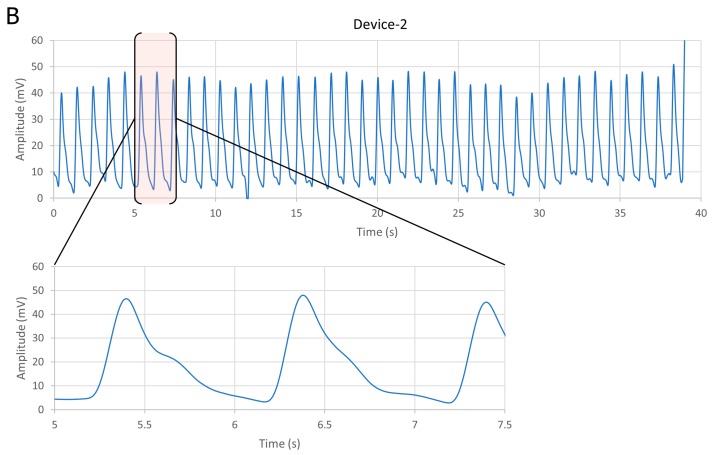
Obtaining the PPG signal at a constant voltage source from (**A**) Device-1 and (**B**) Device-2.

**Figure 9:**
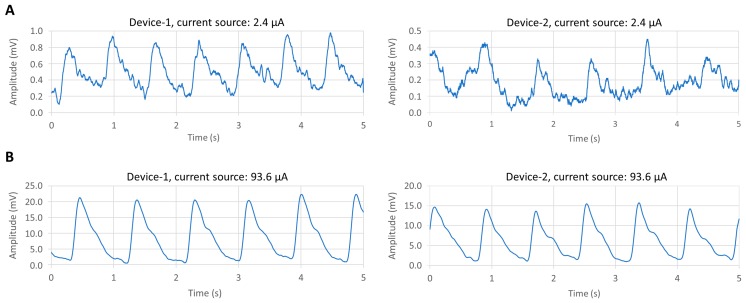
Comparison between the PPG signals from both devices at two different current sources for OLED (**A**) at 2.4 μA and (**B**) at 93.6 μA.

The two pulse meters were evaluated in vivo on a healthy male subject, on his index finger. The PPG signal was collected from Device-1 and Device-2 sequentially, within a specific time period, while he was sitting on a chair. The data were recorded at the sampling frequency of 500 SPS, 8-bit resolution from the SCB and used to evaluate both devices. Figure 8 shows that both devices were reliable and obtained a clear and stable PPG signal. With Device-1, Figure 8A, the peak-to-peak amplitude (V_pp_) of the PPG signal was about 100 mV, while in Figure 8B, the PPG amplitude of Device-2 was less than 45 mV with a constant voltage of 5 V of the OLED. Consequently, the power consumption of each device was different. Device-1 consumed about 1.6 mW and Device-2 about 0.6 mW. Therefore, in order to unify the power consumption of both devices, we supplied a constant current. We compared the amplitude and signal-to-noise ratio (SNR) values of both devices for each PPG signal at different OLED’s driving currents, from 1.2 μA to 93.6 μA, as shown in Figure 9. The constant current was supplied using the MCU’s analog current source. Table 2 summarizes the PPG signal characteristics of Device-1 and Device-2. The method for quantifying the PPG signal quality using the SNR measurement was previously described in [17].

**Figure 10:**
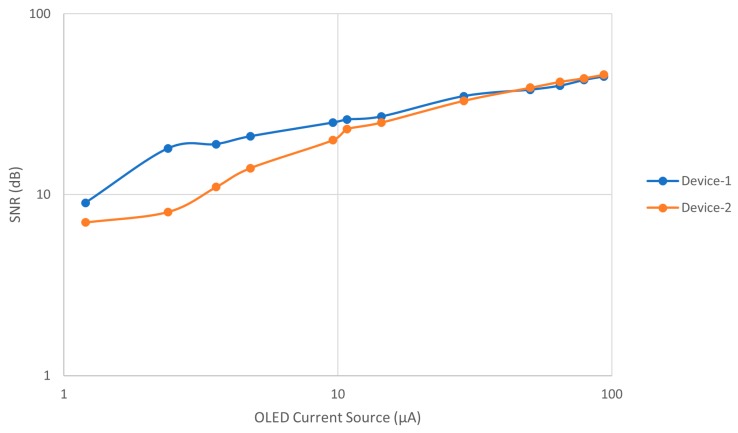
Comparison of the signal-to-noise ratio (SNR) of the PPG signals from Device-1 and Device-2 at different OLED driving currents from 1.2 μA to 93.6 μA.

Figure 10 compares the SNR values from Device-1 and Device 2 of different gap distance at different current consumptions. It shows that decreasing the distance of the gap between the OLED and the OPD, in Device-1, leads to increasing in the DC noise on the OPD, as was indicated by the SNR results with respect to the OLED current. The SNR of Device-2 at a higher OLED current of 93.6 μA was 45 dB, whereas it was 46 dB in Device-1 at the same current source even though its PPG signal amplitude was smaller than Device-2. On the other hand, reducing the gap between the OLED and the OPD resulted in a higher SNR of the PPG signal at a lower OLED current. As shown in Table 2, the PPG signal amplitude increased from 0.3 mV at 8 dB in Device-2 to 0.7 mV at 18 dB in Device-1, where the gap distances between the OLED and the OPDs were 1.65 mm and 2 mm for Device-1 and Device-2, respectively. It is worth mentioning that Device-1 produced an acceptable PPG signal of about 18 dB with ultra-low power consumption, as low as 8 μW. Consequently, from these results, Device-1 demonstrated a significantly advantageous SNR at a low current supply compared to Device-2 where the short gap between the OLED and the OPD increased the chance of obtaining more reflected light. That means the shorter gap between the OLED and the OPD devices will result in improving the SNR at the low power consumption, and the larger gap will result in reducing the SNR.

Although the gap distance between the OLED and the OPD is a top priority while designing an organic pulse meter, another two aspects that should be taken into consideration are the OLED’s area, and OPD’s area. For the OLED’s area, if we reduced the OLED’s area in order to reduce the power consumption, the OLED’s lifetime will also be reduced because the current density will be increased on a small area. On the other hand, if the OLED’s area increased, then the area in the center of the OLED will be impractical and will not contribute to increasing the reflected light from the human body. For the OPD’s area, it should be designed in a way to surround the OLED sufficiently in order to collect more reflected light. However, increasing the OPD’s area too much will result in increasing the DC noise. Therefore, the area of the OPD should be consistent with OLED design, which can be achieved by optical simulation.

Table 3 compares the results between our device and other devices, despite the fact that the previous PPG signal monitoring systems are different than our current proposed device in terms of device characteristics, flexibility and signal quality. The proposed device consumes the lowest power for light source among them.

### 3.2. Results of BLE PPG Signal from Device-1

This section presents the results of the wireless PPG waveforms from Device-1, which was selected as the more effective device, in terms of power consumption, for the portable pulse meter. The PPG signal was obtained from the index finger of the subject while he was resting in a chair during a specific period of time. The data were obtained wirelessly from the portable organic pulse meter using BLE technology. The PPG signal was sent to a PC host using a universal serial bus (USB) dongle (PRoC BLE CYBL10162-56LQXI, Cypress Semiconductor, San Jose, CA, USA) with 500 SPS of ADC and 8-bit resolution. The recorded length of the PPG signal on the receiving PC host was constrained by the sampling rate of the ADC in the chip. Although the BLE chip had an on-air data rate of 1000 kbps, the maximum throughput data rate between the chip and our receiving PC host was 256 kbps at a minimum connection interval of 7.5 ms. The maximum throughput was measured using the PSoC Creator program from [26] on the BLE module and displayed on terminal window software (Tera Term, Ver. 4.93).

**Figure 11:**
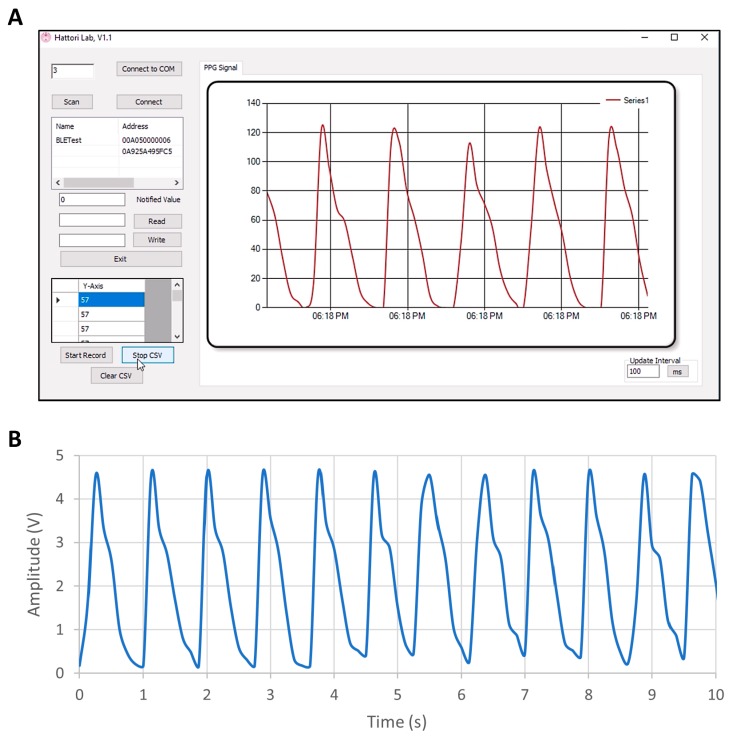
(**A**) The C# program shows PPG signal obtained from a universal serial bus (USB) dongle that was connected to the portable pulse meter via BLE. (**B**) PPG waveform after being recorded from the program as a comma-separated values (CSV) file.

Device-1 successfully showed a very clear PPG signal on the receiving PC host. Figure 11A shows the wireless PPG waveform on our C# program where the data was obtained from the USB dongle that supported BLE. The role of the USB dongle is to convert the Bluetooth signal to a COM port serial signal. Therefore, the dongle was needed in the case that the PC did not have a built-in Bluetooth or did not support the BLE. PPG waveform was recorded from the program as a comma-separated values (CSV) file and is presented in Figure 11B. It is noteworthy to mention that the PPG signals encounter several factors that can influence the signal’s quality such as the amount of pressure from the human body against OLED/OPD device substrate. The device substrate should be coupled with the skin in order to receive the reflected light on the OPD. However, more pressure on the skin results in more pressure on the blood vessels and that causes weakness of the PPG signal’s amplitude. Therefore, moderate pressure from the human body should be applied to the pulse meter’s substrate.

## 4. Conclusions

This paper compared two structural designs of organic pulse meters, Device-1 and Device-2, in order to minimize the power consumption in wireless monitoring of PPG waveforms. We discussed the optical simulation results of both devices. The proposed designs were simulated using ray-tracing and Monte Carlo methods to evaluate the effect of increasing the light source area and decreasing the gap distance between the OLED and OPD. Based on the simulation, the total optical power of Device-1 was more than double to that of Device-2, where the gap distance between the OLED and OPD was 2 mm and 1.62 mm for Device-1 and Device-2, respectively. The simulation results were verified by fabricating two pulse meter structures with different circular OLED areas at the center of the device but surrounded by same ring-shaped OPD area. The performance of the proposed devices was tested in vivo on a healthy individual. In the experimental results, reducing the gap between the OLED and the OPDs resulted in a higher SNR of the PPG signal at a low OLED power source, and a slightly lower SNR at a high OLED power source due to the DC noise. The biosensor pulse meter showed promising results with ultra-low power consumption, 8 µW at 18 dB SNR, and demonstrated its ability to measure a clear PPG signal up to 46 dB SNR at constant current of 93.6 µA. The proposed reflectance-based organic pulse meter sensor was used to wirelessly monitor the PPG signals, and its compatible characteristics were successfully demonstrated. Clear PPG waveforms were obtained from the portable pulse meter via BLE at 500 SPS and 8-bit resolution on the receiving PC host. The maximum throughput data rate between the chip and the PC host was 256 kbps at the minimum connection interval of 7.5 ms. Our proposed device was capable of producing a clear PPG signal and operating on ultra-low power, which is essential for long-term wireless PPG signal monitoring. In future work, the organic optoelectronic device, OPD/OLED, will be fabricated onto a flexible substrate in order to add flexibility to the pulse meter as well as comfortability of use as a wearable medical device.

## Figures and Tables

**Table 1:** Summary of the approximated optical parameters of a human finger.

Tissue	Wave-length (nm)	Index of Refraction (n)	Henyey–Greenstein (g)	Absorption Coefficient (Ua) in mm^−1^	Scatter Coefficient (Us) in mm^−1^	Thickness (mm)
Human Skin	625	1.55	0.81	0.27	18.7	2
Subcutaneous Fat	625	1.44	0.9	1.14	12.8	2.5
Muscle	625	1.37	0.9	0.56	64.7	3
Bone	625	1.37	0.9	0.04	19.5	6

**Table 2:** Summary of the PPG signal quality for Device-1 and Device-2.

Device No.	Average V_pp_ (mV)	SNR (dB)	Current Source (μA)
Device-1	20	45	93.6
Device-2	13	46	93.6
Device-1	0.7	18	2.4
Device-2	0.3	8	2.4

**Table 3:** Comparison table between our device and other devices.

	This Work	Reference [11]	Reference [17]	Reference [10]	Reference [9]
**OLED Type**	Red OLED	Red OLED	Red OLED	Red PLED	Red OLED
**Device Flexibility**	Rigid	Flexible	Rigid	Flexible	Rigid
**Voltage Supply (V)**	3.3	3.3	5	5	9
**OLED Driving Current (μA)**	2.4	21	20	1000	20000
**OLED Area (mm^2^)**	6	0.5	3	N.C.	4
**Power Consumption (μW)**	8	24	100	N.C.	N.C.
**PPG Signal-to-Noise Ratio (dB)**	18	N.C.	45	N.C.	N.C.
